# The International Classification of Functioning Disability and Health Framework (ICF): a new approach to enhance sport and physical activity participation among people with disabilities in Scotland

**DOI:** 10.3389/fspor.2024.1225198

**Published:** 2024-03-15

**Authors:** Liz Carlin, Gayle McPherson, Richard Davison

**Affiliations:** Centre for Culture Sport and Events, University of the West of Scotland, Paisley, United Kingdom

**Keywords:** disability, sport, physical activity, participation, parasport, ICF (International Classification of Functioning Disability and Health)

## Abstract

This research provides a pilot study of the International Classification of Functioning Disability and Health Framework (ICF) involving persons with disabilities (PWD) with and without lived experience of sport participation in Scotland. National surveys in Scotland provide limited information on the nature of individual disability restricting the understanding of the relationship between disability and sport and physical activity participation. The ICF is a framework that aims to describe and classify functioning and thus can be used as a tool to provide a more detailed description of impairment for PWDs beyond their clinical condition. This knowledge has the potential to enhance the development of policies to increase the participation levels in this group. The ICF has also been used to inform the current IPC classification system at a competitive and elite level. As part of a larger study, 450 participants aged between 12 and 70 years completed an online questionnaire examining attitudes to, and participation in, sport and physical activity as well as completing the structural and functional components of the ICF. Subsequently, 18 people participated in focus groups aged between 13 and 61 years. The focus groups examined four meta-theme areas: physical, social, psychological, and sport-specific factors. The results confirm that the ICF provided a more detailed indicator of the key impairments that could have an impact on sport and physical activity participation. There was a clear lack of awareness of the links between the ICF and the classification system for competitive parasport. We concluded that a modified ICF-based assessment tool, incorporating social and environmental factors, has the potential to predict the likelihood of participation and offers a more comprehensive picture of both individual and national disability characteristics. This allows for the development of targeted policies and strategies to assist those with a disability to participate in sport. The overall framework presents a shift in thinking, in policy terms, for those in public health and in sport governance and delivery. The significance of this work is especially concerned with public health and wellbeing and sport development policy as pathways from recreational sport user to elite athlete parasport classification and performance.

## Introduction

This research was part of a larger wide-ranging investigative study of sport and disability linking experiential insight into policy and practice enabling the suggestion of strategic options for improved provision. The Davison and McPherson review of sport and disability in Scotland in 2021 highlighted the potential added benefit of conducting research using the International Classification of Functioning, Disability and Health (ICF), and although Scottish Disability Sport (SDS) embraced this approach, the model had not been used in any Scottish national surveys of people with a disability (PWD) in Scotland. This work offers the first, large-scale, bespoke piece of research on people with a disability participating in sport. The research aimed to provide insight into the lived experiences of sport and exercise provision for people with a disability at recreational, club, and elite-level sport and to use that insight to suggest new approaches to increase participation levels and associated health benefits.

The advancement of both recreational and competitive parasports heavily depends on understanding each athlete's capabilities and effectively applying this knowledge in sport and physical activity settings. This research mainly concentrates on acquiring insights using the ICF and exploring how this knowledge can help individuals with disabilities. In addition, the research acknowledges the connection between the ICF and the Paralympic classification system, examining how this relationship impacts athletes’ progression along performance pathways. Our ambitions are framed by the recognition of the complexity and heterogeneity of the disability experience in its relationship with community sport. The significance of this work is especially important to those concerned with public health, wellbeing, and sport development policy, encompassing the spectrum from physical activity, the recreational sport user to the elite athlete, and was designed to elucidate the direction for more detailed and substantive future research.

## Theoretical context

The Biopsychosocial Model of Disability (BMD) is predicated on the social model of disability with the additional feature of accounting for the subjective experiences of persons with a disability. It is based on psychological and medical variables and can contribute to or precipitate from an ability to participate in physical activity (1) presented by social and environmental factors. Highlighting the complexity of disability, the BMD recognises criticisms of the Social Model and the Medical Model and addresses the relationships and interactions between the biological, social, and psychological factors, supporting viewpoints that disability is not a constant but an entity manifested by the situational factors of the individual (2). Furthermore, it understands that disability and the impairments resulting from that disability are not solely created by the social context they are viewed within (3, 4). This model has become one of the main tools used within health services to assess an individual and their needs when making a diagnosis of a disability or a health condition; however, this has not yet been established for all health conditions (4). The addition of COVID-19 has witnessed further isolation from access to physical activity, and the added ableist response that exacerbated the situation in some areas has led to some people with a disability experiencing more mental health problems and the possibility of further physical health problems without access to physical activity.

Understanding disability is not just a right, but that including persons with a disability in everything we do is the right thing to do; it allows us to create accessible buildings, spaces, transport, and social support structures to lead to a more inclusive approach to providing access to physical activity and sport for persons with a disability.

The International Classification of Functioning Disability and Health [ICF (5)] provides a conceptual framework that challenges the perceptions of physical activity as a primarily performance-based measure of participation (6). It recognises health as a multidimensional and multifaceted taxonomy thus requiring the conceptual framework to aid classification, measurement, and the understanding of functioning (7, 8). Drawing from criticisms of previous classification systems such as the International Classification of Impairments Disabilities and Handicap (ICIDH), the ICF seeks to remove any ambiguity and to better highlight the relationship between the constructs of the ICIDH.

While the ICF is the primary system used for assessing and diagnosing disabilities and health conditions, it is important to consider that there are forms of disabilities that are more hidden and can be extremely complicated to diagnose and classify. The classification of learning disabilities and intellectual disabilities can fall into this bracket with both the International Classification of Mental and Behavioural Disorders (ICD-11) (9) and the Diagnostic and Statistical Manual of Mental Disorders [DSM-V-TR (10)] being the two primary systems used by healthcare systems.

The conceptual framework constructed to represent the ICF has been based on the Biopsychosocial Model of Disability which recognises elements of societal, environmental, and individual personal factors; however, it does lack medical factors outlined by the biomedical model, and this is potentially an area that requires further research and evaluation (11). In addition, criticism of the ICF outlines that it is quite vague in how it distinguishes the causality of the relationships between the three constructs (12). We were cognisant of this throughout the research.

Informed by the Biopsychosocial Model of Disability, the ICF provides a measure of disability and impairment measuring an individual's functional ability and how this influences their day-to-day life. The ICF implements key concepts from the Biopsychosocial Model of Disability (13) such as assessing environmental and societal factors as well as physical structure and function and promotes a holistic view of the individual (14).

The factors influencing sport and physical activity participation, including physical, societal, cultural, psychological, and environmental are understood to be complex, interlinked phenomena that are ever evolving in their nature, particularly for those with a disability (15). The multidimensionality of physical activity is inclusive of motor skills, execution, and engagement in tasks (16, 17), which elicit complex interactions dependent on the individuality of participation.

Beyond the health complications related to disability, it is well recognised that individuals with a disability generally have lower levels of physical activity and thus are more susceptible to additional hypokinetic health conditions. For example, both adults (18, 19) and children (20) with a disability have significantly higher rates of obesity than their non-disabled counterparts. Neter et al. (20) reported that significantly more primary school children with a disability played sport less than 1 h per week and or weekend, or not at all, and engaged in more sedentary behaviour than children with no disability.

A strength of the ICF is its ability to take account of individual physical, structural, and functional impairment, challenges, and barriers. These data can then be used to help identify the challenges individuals with a disability face both within day-to-day life (21) and when trying to access sport and physical activity (22), which can improve our understanding of the experiences of people with disabilities. However, what is still unknown is how the different ICF components, e.g., personal factors, physical structure, and function, interact with each other, which limits our overall understanding of individuals’ experiences of living with a disability (23) or taking part in sport and physical activity. Furthermore, the ICF (5) has not been utilised in Scotland to understand the experiences of people with disabilities in day-to-day life or within a physical activity or sporting context, which has been highlighted as a significant gap in the knowledge base (24). The wide use of the ICF taxonomy within sports classification and its adaptability for free use in multiple languages suggests there is possible scope for the development of a sports-specific version to be utilised by coaches in determining the specific needs of their athletes.

While for those at a participatory level, the ICF may prove important to help understand their needs to support participation, at a more competitive level, the ICF has helped shape and inform the classification system required by the International Paralympic Committee (IPC) for competing athletes. Classification aims to use an evidence-based approach to ensure an individual's impairment has minimal impact on the outcome of their sport participation and performance (25). The development of this evidence-based approach is evident from the significant increase in empirical research, which has been highlighted within the conceptualisation of the process for evidence-based classification from the development phase relevant to each sport and impairment type, the translational phase of implementing required changes to systems, and the regular evaluation of this through the monitoring phase (26). The empirical reliability demonstrated within the ICF and indeed the international acceptance of the framework has allowed for the mapping of the structures within the ICF with the domains of Paralympic sport outlined within the IPC position stand on classification (27). Despite the increase in evidence-based research, there is limited evidence seeking to understand the knowledge and experience of athletes and athlete support personnel (ASPs) on the ICF and its relevance to classification. Recent evidence suggests that athletes will learn through observation of others and thus are dependent on the knowledge and understanding of others (28).

## Methodology

The data presented here are part of a larger study designed to gather evidence of the lived experience of individuals with disabilities living in Scotland, focusing specifically on their participation in sport or physical activity based on four key areas:

(1) Demographics to understand the characteristics of participants and, in particular, those taking part in sport and physical activity.
(2) Participation in sport and physical activity including the what, when, where, and why of participation.
(3) Physical functioning and body structures, understanding their disability using the ICF.
(4) Motivations and barriers to participation in sport and physical activity.

Throughout the study, regular consultation took place with a stakeholder group within the Observatory for Sport in Scotland (OSS). This stakeholder group consisted of representatives from organisations, both sporting and non-sporting, involved in supporting, advising, or providing activities to people with disabilities such as sport's governing bodies, advocacy groups, charities, and support groups. Some organisations represented members with specific impairments including hearing or visual impairments or people with intellectual disabilities; however, the majority of organisations included people with a range of disabilities. This group helped inform the development of the project and subsequent questionnaire while also disseminating the final questionnaire to their members and assisting in the promotion of the research via their social media platforms. Due to the breadth of data provided by this study, this paper will focus primarily on research questions 1 and 3 due to their direct relationship to the ICF data. Areas such as classification and participation relevant to research questions 2 and 4 will also be discussed; however, there is no scope within this paper to cover these more fully.

### Participants

A total of 450 people took part in the study completing a large-scale, online questionnaire that was targeted at all people aged 12–70 years living in Scotland who have a disability. Using population statistics and data from the Scottish Health Survey, it was estimated that the maximum population within the age range who would be classed as a sport participant with a disability would be ∼315,000. Inputting these data into a sample size calculator suggested that for our target population, using a 95% CI and 5% margin of error, an ideal sample size would be ∼400 participants. Therefore, the target for our study was 400 participants to hit the ideal sample size; this would also include a sample of people with a disability who did not currently take part in sport as a comparator group. In addition, a series of six focus groups were conducted with a total of 18 participants regarding their lived experiences of participation in sport and physical activity. Within the focus groups, participants ranged from 13 (with a parent present) to 61 years old. Participants included people with physical disabilities (*n* = 15), intellectual disabilities (*n* = 2), and sensory disabilities (one visual impairment and two hearing impairment). The range of physical disabilities and levels of impairment varied among participants.

### Recruitment

Participants were recruited utilising both convenience and snowball sampling. Targeted convenience sampling was initially conducted by sending invitations to take part in the study to eligible participants via our stakeholder organisations included in the study and agreed upon at the outset. This included 10 organisations representing sports governing bodies, disability sport organisations, disability advocacy organisations, and disability support organisations. This ensured we were reaching as wide as possible a range of groups with disabilities rather than leaving to chance and only have some disability groups covered. Stakeholder organisations represented people with all disabilities and all age ranges and were geographically spread throughout Scotland ensuring no disparity based on where potential participants lived. In addition, including non-sporting organisations provided the opportunity to recruit participants who did not take part in sport or physical activity.

To widen the study beyond our stakeholder organisations, further invitations were posted on social media to increase the potential reach of people with disabilities across all areas of Scotland. The invitation included additional requests to share with all relevant contacts of the participant. It was agreed with the stakeholder group that this would allow for the most significant coverage possible and perhaps reach hard to reach groups that were not part of official organisations or advocacy groups but were active on social media.

It is acknowledged that with any sport or physical activity research, there is a higher tendency for people who do take part in sport or physical activity to be attracted to the research and therefore are more likely to have experiences or opinions they wish to share. Social media posts for recruitment highlighted that this research was aimed at all people with a disability regardless of whether or not they participated in any sport or physical activity. It is recognised that conducting research in a specific area where potential participants self-select to take part can be more appealing to those who have experience in that area. While this may be deemed imbalanced, it must be considered that those who take part in sport and physical activity at a recreational, competitive, or elite level will have substantially more experience of participation and subsequent challenges, barriers, or facilitators to participation, which provides a greater depth of data to be discussed. Those with a vested interest in improving an area that directly impacts them are most likely to participate in research on that area.

### Data collection

The questionnaire was developed using the online survey platform QuestionPro to collect a range of quantitative data across four key areas: participant demographics, sport and physical activity participation, motivations and barriers, and disability and impairment. To enable comparability of results with Scottish population data, questions related to participant demographics including gender, age, income, education, and employment used the same wording and ranges as the Scottish National Health Survey. Questions relating to sport and physical activity participation required small modifications primarily to the range of options available within the answers to increase the relevancy to the target population. For example, the question “what sport/sports do you participate in?” had options added that were parasport specific including wheelchair basketball, goalball, and para-cycling. Additional questions were added from those included within the National Surveys to increase the level of knowledge gained on the participation of PWDs in sport or physical activity including the length of time of participation, how many days per week, and where participation occurs: in school (PE or after school club), in a sports club (disability-specific or mainstream), community club, or day centre. As we were collecting data on the environmental, societal, and individual personal factors via other questions modelled on the Scottish national surveys, we decided not to include these elements of the ICF into our questionnaire as it would have resulted in significant repetition and significantly increased the length of the questionnaire. Therefore, the ICF data collection was restricted to the structural and functional impairments of the ICF to make a direct comparison to the alternative methodology used in the Scottish national surveys.

The final questions were designed to determine the key motivations and barriers to participation and were developed from the questions used in the Scottish National Health Survey with some modifications to make them relevant to the target group. This typically involved adding additional motivations and barriers for those with a disability that had been established elsewhere in the literature.

Following initial development, the questionnaire was sent to members of the OSS research stakeholder group for dissemination to a sample of their members for piloting. This pilot study recruited approximately 10–15 participants from a sample of members within the stakeholder groups. The aim of the pilot was to ensure that the wording of the questionnaire was appropriate and accessible for a wide range of disabilities that people experienced. Following the pilot, the questionnaire results were analysed and reviewed by three members of the research team with any necessary adjustments made. In addition, there were some requests that the questionnaire be tailored for a specific stakeholder group with issues unique to their disability. Possible changes and compromises were made to ensure that the questions were applicable to as many of the desired target groups as possible, but this had to be limited otherwise the length and relevance of the questionnaire would have been unwieldy.

Data were collected to provide a greater depth on the popularity of different sports, and physical activity varied across participants. Questioning was based on that within the Scottish National Health Survey: “*What sport do you participate in?*” However, the standard answers within the Scottish National Health Survey were adapted to provide a greater range of inclusivity and to ensure they were more representative of this population. No adapted sports or activities are included in the Health Survey; therefore, additions to the answers were necessary to represent people with a disability more accurately.

A series of six focus groups took place, with 27 people originally signing up to take part; however, due to issues arising on the day due to illness and work or family commitments, 18 people attended the focus groups online via Zoom with calls being recorded for transcription and thematic analysis purposes each lasting 60 min. Two of the focus groups were signed by a British Sign Language (BSL) interpreter to ensure accessibility for people who are deaf or have a hearing impairment, and closed captioning was used in all focus groups. The focus groups investigated four meta-theme areas: physical factors, social factors, psychological factors, and sport-specific factors. This enabled the exploration of a range of sub-themes that have been reported within the body of literature and the previous review of disability conducted by members of the research team (24), which covered legacy, impact, isolation, barriers, motivations, transport, and access. These were framed using an informal discussion approach around the key themes, with one or two facilitators from the research team leading the discussion. Individuals could share their lived experiences of participating in sport with a disability and link that experience in relation to the meta-themes that we had identified and shared with them. Presented below is a summary and analysis of some of the key issues from the discussions that are useful for understanding the policy implications and practice for stakeholders, whether that is at the national and local governmental level or venue and club provision level.

### Data analysis

Following the completion of the survey, a process of data cleaning and coding was undertaken. The Statistical Package for the Social Sciences (SPSS v29) software was used to complete a series of descriptive and inferential statistical analyses on the resulting coded data.

A process of thematic analysis based on Braun’s and Clarke’s (29) six steps of thematic analysis was used to develop the key themes from the focus group transcripts. These were reviewed by two members of the research team for consistency and accuracy, and a further round of review took place after the first focus group to ensure they were appropriate.

### Ethical considerations

This research, tools, and consent and information forms were all submitted through the University School ethics committee. As we were dealing with both adults and children, prior approval from individuals and guardians was sought. This was granted approval in December 2021 and issued with approval number 17252. Consent was obtained online via a series of statements before completion of the survey. Additional statements and conditions were outlined within the questionnaire to ensure accessibility of the survey to those under 18 requiring parental consent or those who lack the capacity or functioning to complete the survey, thus allowing completion by proxy. These statements were guided by The Adults with Incapacity (Scotland) Act 2000, which outlines that research with adults incapable of consenting is authorised under several circumstances. Such conditions include when the research entails little or no risk or discomfort, the person is not objecting, and that consent has been obtained from a person with relevant powers. Consent may be obtained from a legal guardian or nearest relative if no one has been appointed.

## Results and discussion

Given the work carried out for this study and the enormity of the data gathered, we have sought in this paper to use the data from the questionnaire that focus primarily on the results from the ICF questions with additional results reported elsewhere (30). While the focus groups did not specifically refer to the ICF, relevant discussions have been included here to add depth and further understanding of the experiences of participants. This has allowed us to delve deeper into the issues around demographics, sport participation, physical function, and structural impairment and the ICF; within the focus groups, participants raised issues around their personal circumstances and environment relevant to the ICF as well as discussing issues around classification.

### Demographics

Survey respondents represented more males (51.8%) than females (47.1%), with a small proportion preferring not to say (1.2%). This compares with the overall Scottish population statistics of 48.8% male and 51.2% female. However, it is important to note that the distribution of disability in Scotland estimated from the Scottish Health Survey is 43.4% male and 56.6% female (31). In addition, the gender ratio for those who take part in sport and have a disability is 40.5% male and 59.5% female; therefore, although reasonably balanced, our sample would seem to have a slight over-representation of males with a disability compared to the Scottish population. A representation of a range of physical, intellectual, and sensory disabilities was provided within the survey due to the recruitment strategy and support of the OSS stakeholder advisory group. However, the numbers of participants with sensory, visual, or hearing impairments were lower than the population prevalence indicating a greater need to engage further with people with visual or hearing impairments to ensure that their lived experience is understood.

The sample included a higher than expected representation of 16–24 year olds and a lower than representative sample of the 41–48 years age group. The higher representation at the younger age groups suggests a link with the growth in opportunities and accessibility of disability and adapted sport and physical activity and participation.

**Figure 1:**
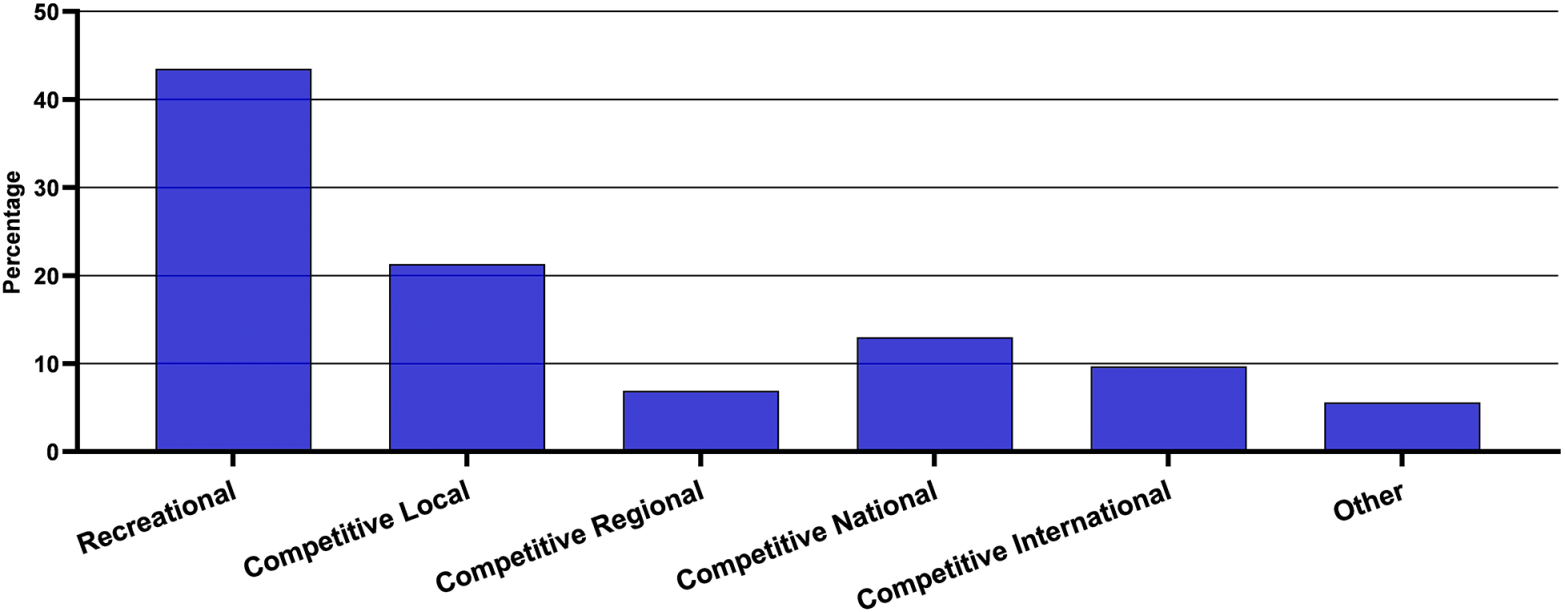
Level of sport participation.

Overall, in our sample, 83.4% of the respondents took part in sport or physical activity. This is significantly higher than the most recent UK Annual Disability and Activity Survey, which reported an inactivity proportion of 41% for 2021–2022 (32). Within our sample, there were significantly more males (88.6%) taking part in sport and physical activity than females (76.5%). The level of participation illustrated in Figure 1 shows that most of the sport participation was for non-competitive recreational (43.5%) reasons. In terms of volume of sport and physical activity, the sample exercised on average 3.83 ± 1.97 days per week, and when asked how much time on one of those days, this ranged from 30 to 300 min with an average of 102 ± 58 min. This suggests that for this sample, a significant proportion easily exceeded the UK Chief Medical Officers recommendations of at least 150 min of moderate-intensity activity or 75 min of vigorous activity per week (33). However, closer examination revealed that 22.4% of our sample failed to reach the 150 min of moderate activity per week.

Most of the respondents took part in sport/physical activity both in a group and on their own (55.7%) with equal proportions on their own (20.8%) and in a group (23.5%) only. This would suggest that flexibility in the opportunity to exercise on your own and in a group is important to increase participation. We found no link between gender and participation suggesting that there was no difference between the genders in terms of whether they took part in sport and physical activity in a group, on their own, or both.

In terms of activities undertaken by the sample, walking or the equivalent of wheelchair pushing topped the list of activities at 14.2%, followed by swimming (12.7%) and gym/weight training (7.5%). The “other” category, which comprised 9.7% of responses, included various sports such as horse riding, judo, karate, hockey, kayaking, petanque, indoor climbing, cricket, sailing, wheelchair boxing, shinty, Tai Chi, Taekwondo, darts, water polo, trampolining, pilates, and rowing. These data show the diverse range of sports that are currently being practiced, and similar to trends in non-disabled sport participation (34), there appears to be a predominance of individual sports over team sports. While a large proportion of the sample participated at a recreational level (43.5%), over 50% were engaging in competitive sport and over 20% were competing at the national or international level. There were 20% of participants competing at a local level, and this fits well with Scottish Disability Sport's pathway programme, Playground to Podium, and their model of inspiring through inclusion. Part of their 2021–2029 Strategic Plan highlights that 80% of people with a disability want to take part in more sport and physical activity (35), but that 47% of individuals were concerned about losing their benefits if they were seen to be physically active. This was also raised in our focus group discussions as a real fear by many and certainly a cause for concern when thinking about taking to a more competitive level. It is important to consider the disconnect between the message of seeking a more active population yet having a benefits system where the level of mobility of the individual directly impacts the overall assessments of the level of financial support they may require.

### ICF

Participants completed the physical functioning and body structure components of the ICF using a 5-point Likert scale with responses of “no impairment, mild impairment, moderate impairment, severe impairment, and complete impairment.” These responses reflect the standardised responses of the ICF to ensure accurate and comparable reporting of results.

**Figure 2:**
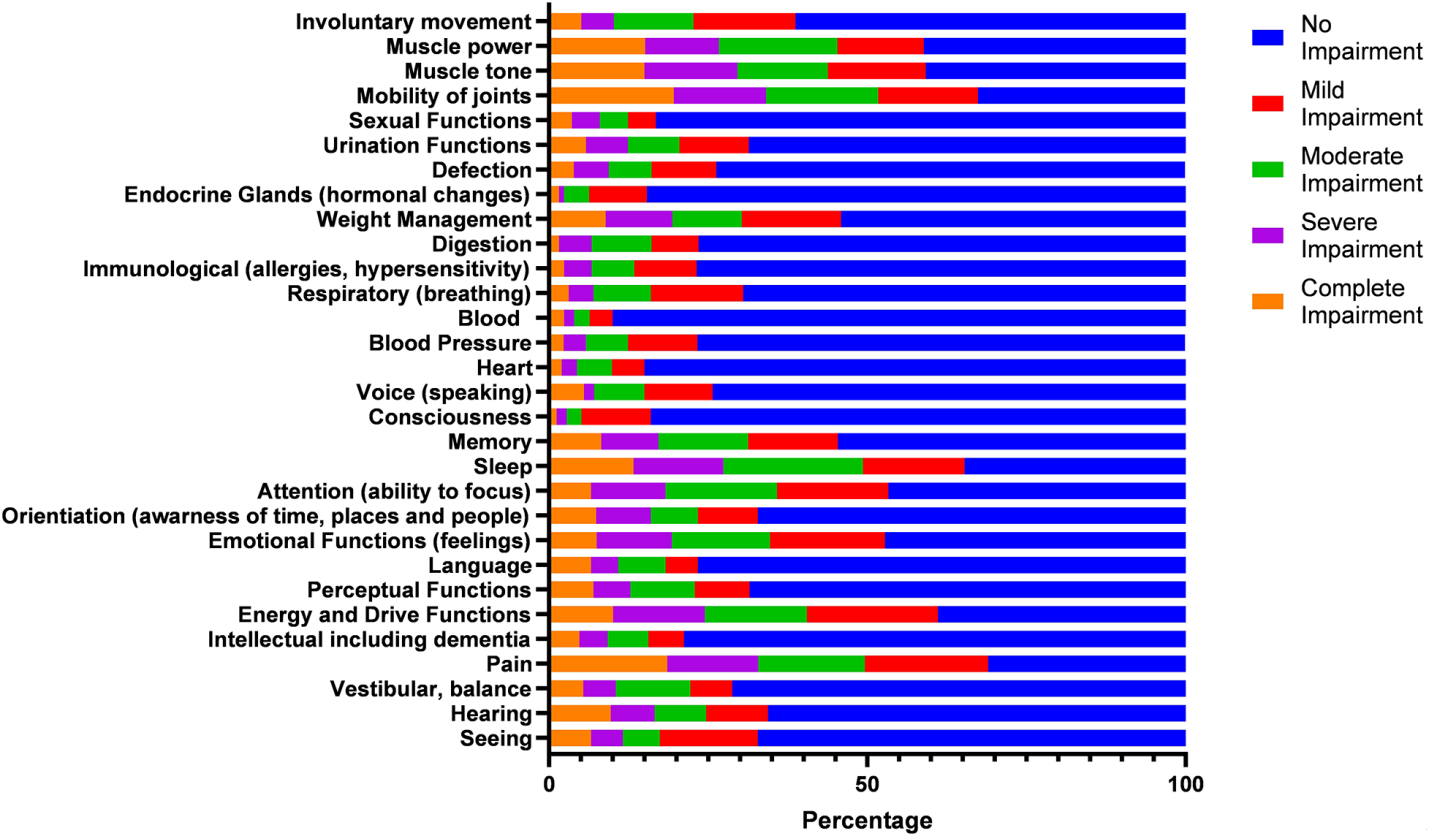
ICF measures of physical function.

The ICF measures of physical function refer to the physiological and psychological functions and structures of the body such as sensory functions, mobility, and communication. Figure 2 indicates the range of physical functions included within the self-reported measurements including mobility, involuntary movement, muscle power and tone, endocrine glands (hormonal changes), respiratory, blood pressure, heart, intellectual, pain, hearing, and seeing.

The most widely reported physical function was pain with 69% of respondents indicating an impairment, mobility of joints (67.4%), sleep (65.3%), muscle power (58.9%), muscle tone (59.2%), energy and drive functions (61.1%), and emotional functions (feelings) (52.8%). These include all impairments ranging from mild to complete. The least reported physical function impairments were consciousness (16%), heart (15%), and blood (10%) with the majority of these stating a mild impairment. The most commonly reported physical functions with complete impairment were pain (18.6%), mobility of joints (19.6%), muscle power (15.1%), and muscle tone (15%).

Within competitive parasport, athletes must undergo a process of classification that determines the eligibility to compete alongside athletes with similar impairment types or levels. These may vary between sports, for example, within Boccia, athletes may be classified as BC1–BC4, which determines the level of assistance or use of a ramp or aid that the athlete may use. This classification process is informed in part by the physical function and structural impairment data similar to the data collected in the ICF. The knowledge this provides for individual athletes at a club level can influence talent development and pathway models within Sports Governing Bodies and Scottish Disability Sport seeking to grow elite-level sport and grassroots participation. If this process is understood at an earlier level within clubs, the correct provision and support can be afforded to each person to develop to the level of competition they are aspiring to or indeed enable them to focus on participation if that is their preference.

**Figure 3:**
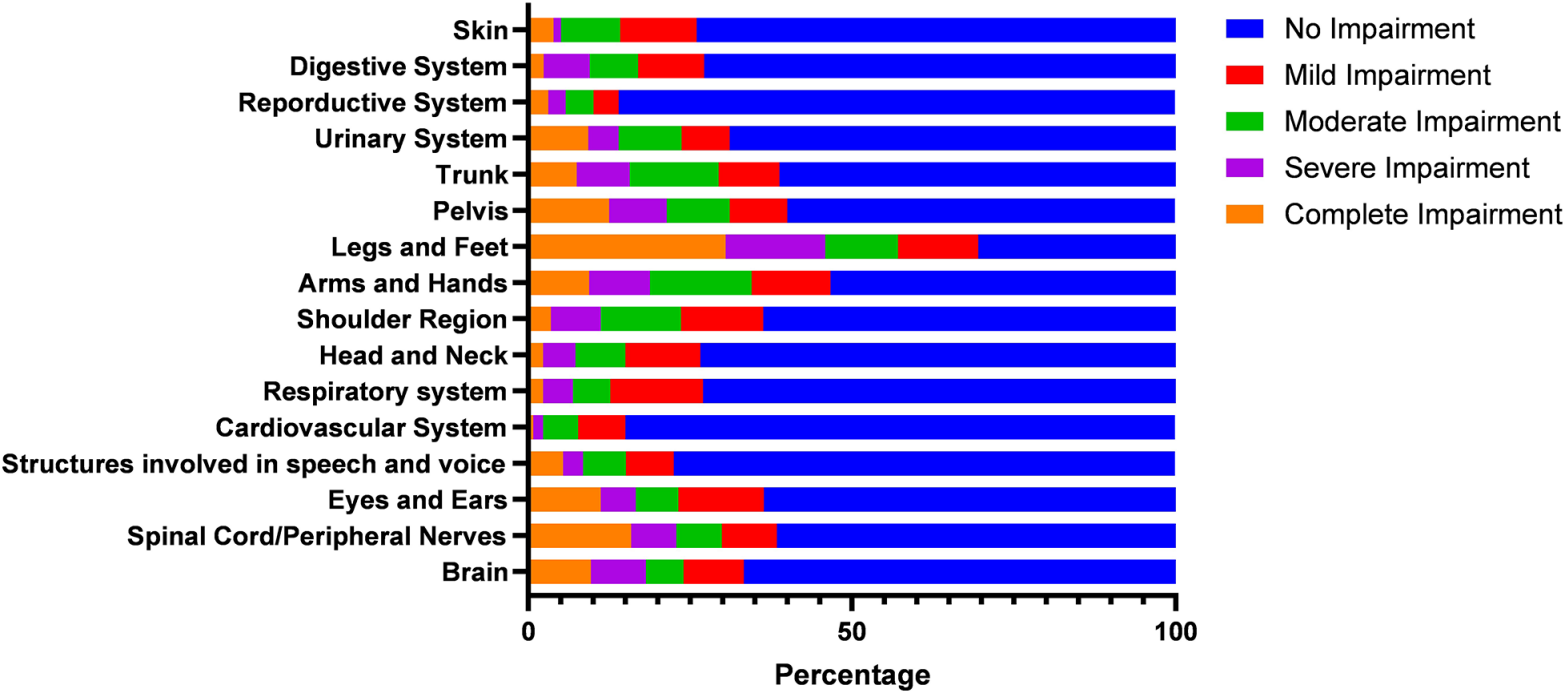
ICF measures of structural impairment.

Figure 3 reports the data on the structural impairments included within this section of the ICF. They include legs and feet (69.5%), spinal cord/peripheral nerves (38.4%), eyes and ears (36.4), arms and hands (46.7%), trunk (38.8%), pelvis (40%), and the shoulder region (36.3%), among others. The highest reported levels of complete impairment (30.5%) were legs and feet, with reproductive systems (14%) and the cardiovascular system (15%) showing the lowest overall impairments, while the cardiovascular system also had the lowest rate of complete impairment (0.8%).

It is interesting to note that lower body structural impairments and functional impairments related to mobility, muscle tone and power, and pain are among the most significant challenges for this group of participants. These findings suggest that interventions aimed at improving mobility, reducing muscle tone and power impairments, and managing pain could be particularly beneficial for this group in terms of increasing their participation in physical activity. It is also useful to consider the specific impairments most common or severe within this group, as this will help inform the development of targeted interventions. In addition, it would be helpful to gather more information about the context in which these impairments are experienced, such as the type and intensity of physical activity attempted. This granular detail that allows for a more targeted approach will help ensure specific information for providers of sport and physical activity opportunities. This also provides policymakers with specific data on the level of impairments, not previously known, and can be used to help target funding and strategic developments towards specific impairments and inclusion of marginalised groups. From the ICF question responses, it is possible to generate an individual ICF score, which in conjunction with other data could be used to establish relationships and associations that would not be possible with the standard assessment of disability status used by the Scottish national surveys. In this survey data, the respondents who indicated that they did not currently take part in sport and physical activity had a significantly higher ICF score (91 vs. 78) suggesting that these individuals had a significantly higher level of disability. This would seem like an obvious relationship, but to date it has not been possible to measure the relationship between the level of disability and participation. It also adds a new variable that can be used in conjunction with others to determine the most important factors influencing sport and physical activity participation. Bearing in mind disability is often only one of many factors that affect a PWD and understanding the wider environment and detailed structural impairments of people would help with wider social integration.

### Classification

As many para-athlete classification systems are based on the ICF, we specifically sought the views of participants of the focus groups on the classification systems. Knowledge of the process of classification was limited with the majority of participants indicating this was only relevant for those with an interest in competitive or elite-level sport. However, it was indicated by some participants that this did discourage them from seeking classification and thus taking part in competitive sport. While participants acknowledged that their understanding was limited in relation to the process of classification, there was a discussion of how this could benefit them. Limited support was available for this from coaches who equally had limited understanding and were therefore unable to provide guidance and advice leading some individuals to make assumptions on the lack of relevance for them. The importance and influence of classification on perceptions of ability to participate provides further support for research indicating this is a disability-specific barrier (36) and subsequently the need for a clearer and more simple process. Within the context of Scottish sport, the use and knowledge of the ICF are limited with this study providing the first large-scale research utilising the ICF in Scotland and supports the work of Lawson et al. (28) highlighting the need for a greater understanding of the ICF and the classification process among athletes. More effective utilisation of the ICF at a participation level and the increasing awareness among coaches and people with disabilities could help bridge the gap between physical activity, recreational participants, and those competing at a level requiring classification. Within current club and governing body structures, the ICF could inform initial pathways to participation by highlighting the characteristics and strengths of individuals suited to particular parasports. However, the standard ICF as used in this study is potentially complex and unwieldy to be easily applied at participation and local sporting levels; thus, we would recommend the development of a sport-specific version of the ICF, which could inform both coaching and participation and may indeed inform talent identification within parasport structures.

Current issues identified with the parasport classification systems highlighted in the focus groups should be considered in the development of a modified ICF version based on sport and physical activity. Overall classification was a highly emotive issue and one that those in the groups felt needed to be addressed by those at a policy level in sport and for the Scottish Government to understand the importance of such decisions made for elite athletes representing their country; and in their potential to act as ambassadors abroad. Discussion items in our focus groups centred around cheating to secure a classification and not being seen as disabled enough in some categories to compete. The re-definition of classifications was mentioned, and there was a lot of confusion over why some sports and classifications needed so much adjustment. Levels of equity and fairness as well as the emotional distress this caused for the athletes were highlighted. For classification, some participants had to “go out with Scotland to get tested,” which increased the travel burden, cost, time, and reliance on family members or support workers to assist with obtaining a classification. Participants affected by this indicated the disparity with some international counterparts such as in Australia, classifications can be assessed and confirmed by video footage as the rural nature of some of the landscape and distances needed to travel would further disadvantage disabled athletes. This was coupled with conversations focusing on accessibility and geography with participants indicating that most opportunities to participate required travel if they lived outside of the central belt in Scotland.

Reflections of stories of teammates or friends considering making life-changing decisions to aid in classification highlighted the severity of the issue with one “even considering getting his leg amputated so that he could meet the criteria” rather than having to give up a sporting opportunity. Consideration of the traumatic nature of the current classification systems and their impact on participants is required. Within some governing bodies, the reporting of athletes without classifications led to disparities in the reporting of the numbers taking part and disabilities hidden. For example, “Although I tick the disability box in the club and (NGB) can say they have so many disabled athletes, when results are published there is nothing that says I am a disabled athlete. I’m not ill enough to get a classification. It's why I like the park run model, I can have 2 clubs, one that says running club and one that says MS.” Increasing visibility and showing that people with MS or CP take part can remove the element of disability being hidden within sport.

### Conceptualisation of the ICF

The data gathered within this study suggest a clear need for an overarching conceptual framework revealing the complexities of the interactions between sport and physical activity participation and people with disabilities. The multifaceted nature of disability highlighted within the Biopsychosocial Model of Disability indicates the necessity to understand these complexities from all levels of influence. The results outline that participation was influenced by factors including social support, facilities, opportunities, infrastructure, and financial support.

**Figure 4:**
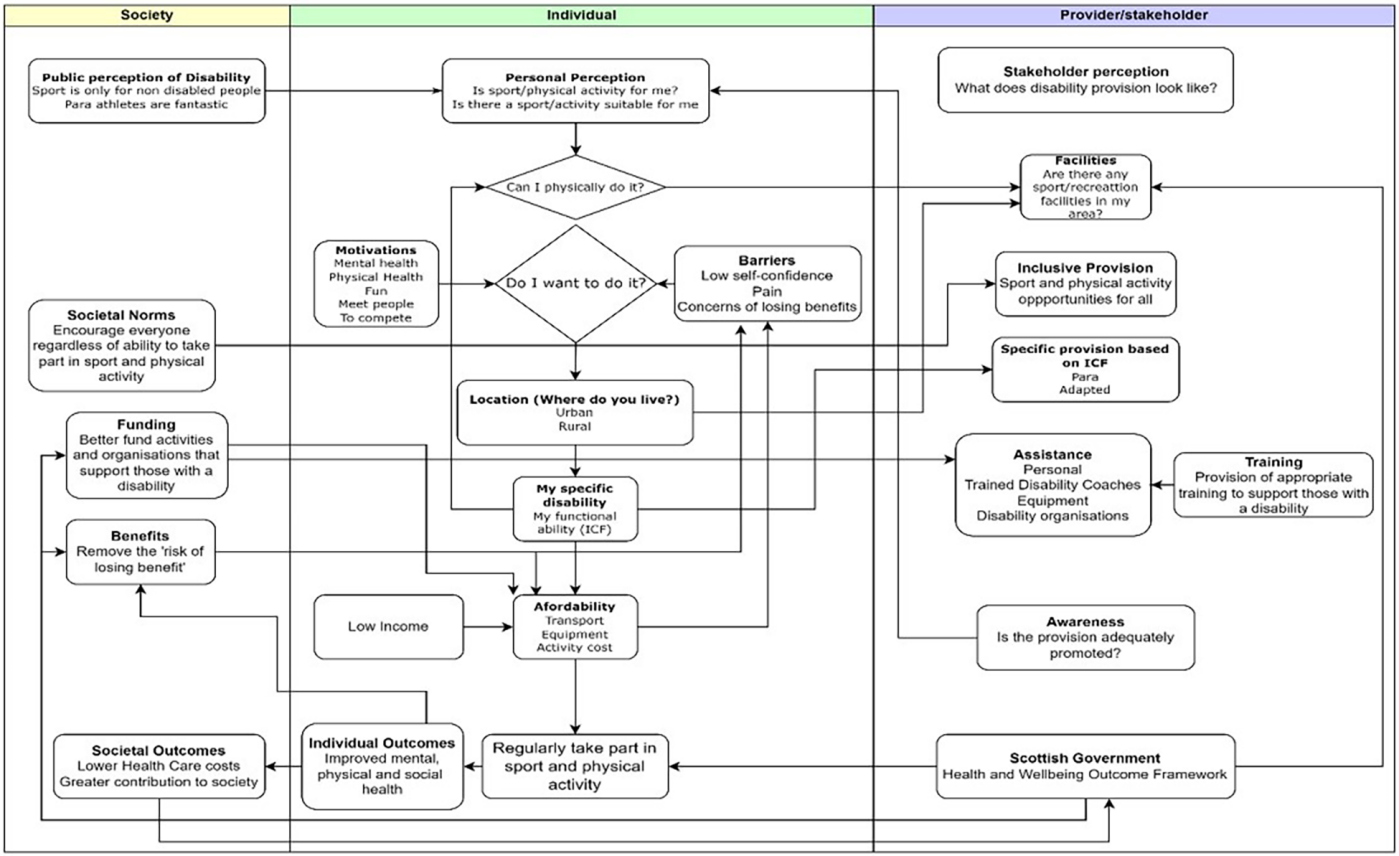
A conceptual framework of micro, meso, and macro influences on sport and physical activity participation for people with disabilities.

Davison and McPherson (24) identified the need for greater connection between those in policy and power positions of sporting governing bodies and with events organising committees to facilitate and demonstrate a flexible person-centred approach to classification, sport by sport. The model in Figure 4 visualises the complexities of the interactions from the micro-, meso-, and macro-level perspectives of the individual, society, and the provider/stakeholder and is useful for individuals, organisations, and policymakers to understand how the overall process of sport participation by PWD and the importance of inclusion in society can be better realised. Central to this model is the individual and their specific disability and the level of functional impairment identified within the ICF. While improvement in inclusion and opportunities has indeed been seen, what must be considered in greater depth is the potential positive influence that the ICF can have in developing knowledge and understanding at the individual level and how the individual level can inform the societal and stakeholder levels.

The interlinking nature of the framework illustrates the range of influences on the participation of people with disabilities in sport and physical activity. Only when a holistic approach is taken to understanding the physical, social, structural, and environmental landscape can we move beyond ableist assumptions of disability (37) and the language used in assessing individuals with a disability. The survey included key personal and contextual components from the ICF including social background, education, race/ethnicity, and environmental factors of attitudes, support networks, and transportation represented within the individual and societal levels of the conceptual framework.

The ICF has been shown to be influential within numerous contexts including physiotherapy and rehabilitation for people post-stroke and within the classification of athletes with disabilities for parasport classification; however, this influence has not yet extended to physical activity and parasport participation. We must query if this is in part due to a lack of understanding of the ICF in grassroots parasport, the lack of a sport-specific version, or the misinterpretation of its relevance outside of an elite parasport context. We would argue that a combination of these factors has played a role and needs to be addressed, and the first stage of this is the development of a sport-specific version of the ICF.

The ICF influences participation from all levels of the framework from the functional ability of the individual at the micro level, societal attitudes and perceptions at the meso level, and inclusive and/or specific provision from organisations and stakeholders at the macro level. The micro level focuses on the disability-specific factors that can influence sport or physical activity participation, the perception of the individual of their ability, and the barriers impacting these. While the ICF can focus on the structural and functional impairments of an individual, a sport-specific version can further the provision of adapted and inclusive sport by providing the coach with a depth of knowledge for each participant.

The implementation of the theoretical and conceptual underpinning of the ICF within strategic policies at a sport governance level would enable a more tailored approach to meeting the complexities of each sport. Linking with the Scottish Government’s National Health Outcomes Framework, understanding the needs of the individual to aid participation in sport and physical activity has been evidenced to demonstrate improved outcomes of physical, psychological, and social health, thus reducing potential costs of health and social care. Appropriate strategy and policy can only be formulated with detailed knowledge of the target population. Thus, with the more detailed information on the exact nature of disability in terms of structure and function, provided by a nationally deployed ICF survey, the Government and other key agencies could develop more targeted interventions and funding to effectively address many of the issues outlined by the participants in this study.

### Potential use of ICF by sport coaches and physical activity coordinators

The survey responses and discussions within the focus groups outlined the need for more qualified coaches, more awareness of coaches, and more understanding of coaches on how to ensure adequate adaptations and provisions were in place to enable participation within their clubs of people with various disabilities. More specifically, participants in the focus group shared experiences of stopping participating within clubs due to feeling like the clubs were not able to provide for their needs. During major games (London, 2012, Glasgow, 2014), it was felt that the clubs were provided with support to encourage participation through additional equipment, support from governing bodies, etc.; however, as time progressed after the games, this support diminished leaving some people with a disability feeling their needs could not be met by the club. Further education and support would provide the skills, knowledge, and awareness for coaches to provide safe and inclusive sessions. While inclusive practice does form elements of current coach education structures, if enhanced by knowledge and potential application of the ICF, coaches will be better equipped to understand the needs of individual participants and how to best coach that individual to achieve their sporting goals. This does point to the need that the development and adoption of a sport-specific ICF must be supplemented with specific coach education into its utilisation to support wider participation.

The lack of coaches with a disability or with the appropriate level of knowledge and awareness of disabilities and adaptations supports work on barriers to participation and raises the importance of issues with policymakers and venue providers to think more inclusively (30, 38–40). It impacts on decisions to participate not only in the first place but also in the development pathways. This is a significant finding for governing bodies of sport and SDS, who support athletes and clubs. SDS indicates that only 2% of the qualified coaching workforce have a disability (41), and this research supports the need for further investment in coach education training programmes for those with a disability. It helps us move away from the ableist model in which we provide inclusive coaching for able-body coaches on how to accommodate athletes with a disability. Limited knowledge of opportunities and the range of adapted and inclusive sports and physical activities available remains a significant barrier for people with disabilities and indicates an increased need for input, advice, and guidance from sports coaches upon initial decision-making to take part. Our survey highlights the variety of opportunities available; however, given the knowledge of opportunities remains a barrier to participation, it is imperative that organisations work in unison to provide people with disabilities, coaches, and sports clubs with the information to appropriately signpost. Signposting of this information may require a greater level of discussion between the person and the coach to ascertain the motivations of the individual, their preferred level of competition/participation, and their individual needs to inform the activity that may be best suited to that person or that they wish to take part in.

The depth of data provided by the ICF is invaluable to sports coaches in determining the needs of the individuals seeking to participate within clubs. However, the current version of the ICF produces a plethora of data that may not directly or indirectly impact the participation in sport and physical activity. The significance and rigour applied in this study to examine the physical function and structural impairment components can aid in understanding the physical adaptations a person may require participating in any activity or the support that may be required to do this. This could be used to help transform disability coach education programmes and elite athlete training needs analysis as part of the pathway approach adopted by SDS. The significance of the environmental and contextual information provided by the full ICF is also recognised and allows for a holistic picture of athlete needs. This study demonstrates the need for further research to develop an ICF sports participation version that will encompass all relevant information for sports clubs and coaches.

Coach education to enhance the understanding of the participation of people with a disability was discussed throughout the focus groups, and the consensus suggested difficulties in accessing coaches with the knowledge to help the individual participate and develop. Utilisation of an ICF sports participation version could provide coaches with the opportunity to work directly with their athlete or prospective athlete/individual to collaboratively ensure that the person can take part in sport or physical activity in the way most suited to them. The need for reform in coach education structures within parasport (42) would allow for the embedding of ICF training to increase awareness and inclusion of people with disabilities in sport and physical activity. Lessons can be learned from the work undertaken within areas including physiotherapy (43) and rehabilitation (44), which have utilised the ICF to aid in understanding the factors impacting participation within sport and physical activity. Rimmer (44) discusses how healthcare professionals can use the ICF to identify functioning and associated participation within community and activity-based settings, while Mitchell (43) identifies how the ICF could be utilised within a physiotherapy setting in the Scottish National Health Service as a means for improving how rehabilitation is tailored to the problems a patient presents with as opposed to a standard approach based on their diagnosis. This approach highlights how a sports-specific version of the ICF could be utilised by sports coaches and sport governing bodies to improve provision on a more individualised basis for participants.

Despite the recognised additional benefits, not everyone wants to play sport and thus an adapted ICF needs to be flexible enough to encompass the needs of organisations and individuals who are tasked with providing more wide-ranging physical activity opportunities. Low physical activity is harmful to health whether an individual has a disability or not, and data from this study and others (45) show that there is a higher risk of low physical activity levels and higher sedentary behaviour for those with a disability. There is also strong evidence of a positive association between physical activity and cardio-metabolic health for disabled adults with physical, intellectual, and mental impairments (45). It is also important to recognise that this relationship works in the opposite direction in that the significant increases in the levels of disability over the last 30 years have primarily been driven by the inclusion of conditions associated with non-communicable diseases (46). Thus appropriate tools to support those with a disability into more active lives and sporting participation are critical to improve the quality of life and provide equal opportunity to participate.

## Conclusion

This study has provided data from the largest representation of people with a disability living in Scotland regarding participation in sport and physical activity with a range of physical, intellectual, and sensory disabilities and across a balanced representation of males and females and all age brackets from 12 to 70 years. Participation at all levels of sport and physical activity from recreational to international performance level was represented within both the survey and the focus groups.

This study represented the first ever research conducted on the use of the ICF in the context of physical activity and sport participation within Scotland. It included key personal and contextual components from the ICF including social background, education, race/ethnicity and environmental factors of attitudes, support networks, and transportation. It raises many areas of future research required while highlighting the potential use of the ICF to inform policy and strategy on embedding the rights of people with disabilities to participate in sport and physical activity in a meaningful way suited to their needs and potential sporting ambitions. Further work on embedding the rights of people with disabilities within sport and physical activity and to continue to develop a framework to influence the development of policy is required.

Despite the respondents of our survey claiming to be active at the recreational level up to the elite-level sport, closer examination still revealed lower than recommended physical activity levels and high levels of sedentary behaviour, commonly found in individuals with a disability. This behaviour is known to contribute to the development of a number of hypokinetic diseases potentially further exacerbating the medical problems experienced by these individuals. Thus, there is a public health imperative to help individuals with a disability to participate in appropriate physical activity and sport to enhance their quality of life. The interlinking nature of the framework presented in Figure 4 illustrates the range of influences on the participation of people with disabilities in sport and physical activity. Only when a holistic approach is taken to understanding the physical, social, structural, and environmental landscape overall can we move beyond ableist assumptions of disability and the language used in assessing individuals with a disability. At the core of this framework is the ICF or the “functional ability” of the individual; thus, a more granular understanding of both population and individual disability characteristics is critical on all other elements of the framework.

This study did identify common patterns of functional and structural impairment for this group using the ICF, which could be viewed as an initial profile of individuals with a disability who had an interest in sport participation. This information does have the potential to inform some decision-making of key stakeholders, but ideally data from a much larger population are required to inform the development of appropriate interventions and provision at a much larger scale.

The full ICF has limitations in that it covers a wide range of functional domains, and not all of these may be the most efficient tool for assessing factors associated with participation in sport and physical activity specifically. However, it is also important to note that disability is a complex and multifaceted concept, and factors such as sleep, nutrition, intellectual capacity, and physical function can all impact an individual's ability to participate in physical activity. Therefore, it is useful to include these domains in a modified slimmed down ICF-based assessment tool, which also incorporates some of the other well-known social and environmental factors to ensure a complete picture of an individual's functioning and disability that is relevant to sport and physical activity participation. Further research would be required to decide on the most influential domains that could be included in a modified ICF. To effectively understand the needs of coaches, physical activity coordinators, and people with disabilities from a modified ICF, a co-productive approach to the development is required.

Overall, the results of this research have provided a significant new evidence base not only for those in sport governing bodies and policy roles, such as SDS, but also for those in government policy positions who run the Scottish Health Survey and also those with the responsibility for the development of physical activity opportunities for “non-sporty” individuals with a disability. Discussions from this research presented in Davison et al. (30) report highlighting the conflict experienced by people with a disability seeking to be more active; yet, feeling they were risking being deemed “too active” within the current welfare structures emphasises the need for constructive engagement within the political field. The potential of the ICF to inform policy on how functional impairments can continue to impact the daily life of an individual with a disability regardless of their level of physical activity must be further explored. This study has provided a more detailed analysis of the types of challenges and opportunities for development and provision that could be made available for those with a disability that wish to pursue sport participation either at a recreational level or a regional and national elite parasport pathway development programme, thus leading to more inclusive growth and integration of people with a disability in society.

## Data Availability

The raw data supporting the conclusions of this article will be made available by the authors, without undue reservation.
